# Hospital-to-home transitions for children with medical complexity: part 1, a systematic review of reported outcomes

**DOI:** 10.1007/s00431-023-05050-9

**Published:** 2023-06-15

**Authors:** Annemieke de Lange, Mattijs W. Alsem, Heleen N. Haspels, Clara D. M. van Karnebeek, Job B. M. van Woensel, Faridi S. Etten–Jamaludin, Jolanda M. Maaskant

**Affiliations:** 1 Department of Pediatrics, Amsterdam UMC location University of Amsterdam, Emma Children’s Hospital, Meibergdreef 9, Amsterdam, the Netherlands; 2 Department of Rehabilitation, Amsterdam UMC location University of Amsterdam, Amsterdam Movement Sciences, Meibergdreef 9, Amsterdam, the Netherlands; 3 Department of Pediatric and Neonatal Intensive Care, Division of Pediatric Intensive Care, ErasmusMC-Sophia Children’s Hospital, Rotterdam, the Netherlands; 4 Department of Pediatrics and Human Genetics, Emma Center for Personalized Medicine, Amsterdam UMC location University of Amsterdam, Emma Children’s Hospital, Amsterdam Reproduction and Development, Meibergdreef 9, Amsterdam, the Netherlands; 5 Medical Library, Amsterdam UMC location University of Amsterdam, Meibergdreef 9, Amsterdam, the Netherlands

**Keywords:** Transitional care - children with medical complexity, Core outcome set, Systematic review, Reported outcomes

## Abstract

Outcome selection to evaluate interventions to support a successful transition from hospital to home of children with medical complexity (CMC) may be difficult due to the variety in available outcomes. To support researchers in outcome selection, this systematic review aimed to summarize and categorize outcomes currently reported in publications evaluating the effectiveness of hospital-to-home transitional care interventions for CMC. We searched the following databases: Medline, Embase, Cochrane library, CINAHL, PsychInfo, and Web of Science for studies published between 1 January 2010 and 15 March 2023. Two reviewers independently screened the articles and extracted the data with a focus on the outcomes. Our research group extensively discussed the outcome list to identify those with similar definitions, wording or meaning. Consensus meetings were organized to discuss disagreements, and to summarize and categorize the data. We identified 50 studies that reported in total 172 outcomes. Consensus was reached on 25 unique outcomes that were assigned to six outcome domains: mortality and survival, physical health, life impact (the impact on functioning, quality of life, delivery of care and personal circumstances), resource use, adverse events, and others. Most frequently studied outcomes reflected life impact and resource use. Apart from the heterogeneity in outcomes, we also found heterogeneity in designs, data sources, and measurement tools used to evaluate the outcomes.

*    Conclusion*: This systematic review provides a categorized overview of outcomes that may be used to evaluate interventions to improve hospital-to-home transition for CMC. The results can be used in the development of a core outcome set transitional care for CMC.

**Supplementary Information:**

The online version contains supplementary material available at 10.1007/s00431-023-05050-9.

**Table:** 

**What is Known:** *• Studies on the effectiveness of interventions to support the hospital-to-home transition of CMC are numerous*. *•Heterogeneity in outcomes hamper comparisons across studies and therewith the ability to move research forward*.	
**What is New:** *•This systematic review summarizes and categorizes outcomes reported in publications that evaluated interventions to improve the hospital-to-home transition for CMC*. *•In total 172 reported outcomes were summarized to 25 unique outcomes that were assigned to six outcome domains*.	

## Introduction

Hospital-to-home care for children with medical complexity (CMC) and their families is an expanding research area. This is motivated by the substantial increase in numbers of CMC, resulting from medical developments and consequently in the improved life expectancy of children with previously considered untreatable diseases [1, 2]. CMC are defined as children with concurrent chronic conditions, family-identified service needs, functional limitations and high healthcare use [3]. CMC consists of a diverse group of children (e.g. children with severe cerebral palsy or metabolic diseases), and are characterized by frequent emergency department visits, and lengthy and complicated (re)hospitalizations that create pressure on the healthcare system [4, 5]. However, the length of hospital stay of CMC is decreasing [6], and the complex care, such as tracheostomies, enteral feeding tubes, intravenous infusions, dialysis, and complex medication regimens is often provided by families at home [7]. Despite the benefits of being home, caring for CMC is challenging for families, and experiences of parents reveal emotional, social and financial hardships [8–10].

The transition from hospital to home of CMC should only take place when parents feel ready, and when the continuity of care is optimally organized. However, this is not always successful [11], and parents do not always feel supported and adequately prepared [12, 13]. Publications on the effectiveness of interventions to support a successful transition from hospital to home of CMC are numerous, with coordination of care, collaboration between families and the multidisciplinary team, and communication as key elements [14, 15]. However, the heterogeneity in outcomes hamper comparisons across trials and therewith the ability to move research forward in this field [16–18]. An overview of available outcomes may support researchers and program evaluators in outcome selection. The aim of this systematic review was to summarize and categorize outcomes currently reported in publications evaluating the effectiveness of hospital-to-home transitional care interventions for CMC.

## Methods

This systematic review was performed according to the Preferred Reporting Items for Systematic Reviews and Meta-Analyses (PRISMA) guidelines for systematic reviews [19], and the recommendations for a Core Outcome Set development and reporting [20, 21].

We considered this literature review as the first step in developing a Core Outcome Set (COS), an agreed method to overcome problems of variability in outcome selection, measurement and synthesis [22]. The full protocol, describing this systematic review and the next steps (a Delphi study and focus groups), has been registered in the Core Outcome Measures in Effectiveness Trials (COMET) Initiative database [22].

## Study eligibility

### Search strategy

We searched Medline, EMBASE, Cochrane library, CINAHL, PsychInfo and Web of Science with help of a clinical librarian. The search was limited to articles published in English between 1 January 2010 and 15 March 2023. The search was executed on 15 November 2020, and updated on 18 March 2023. This period was considered adequate for the aim of our study as the reported outcomes may change over time depending on e.g. societal perspective on health and healthcare, and population characteristics. The key terms referring to the patient group included “children with medical complexity” or related terms, such as “children with complex chronic conditions”. The key terms referring to the intervention included “transitional care”, “follow-up” and “discharge”. Comparison interventions and outcomes were not specified in the search. The detailed search strategies are presented in Appendix A. Duplicates were removed electronically; the selection was carried out using the web application Rayyan (http://rayyan.qcri.org).

### Inclusion criteria

We included comparative studies evaluating the effectiveness of an intervention to improve the hospital-to-home transition for CMC, e.g. randomized controlled trials, controlled clinical trials, cohort studies, and before-after designs. Studies had to report on quantitative outcomes to be eligible for inclusion. We focused on studies with participants described as children with medical complexity. Studies based in all inpatient settings that provided transitional care were included, as well as all types of interventions or types of care that aimed to improve the hospital-to-home transitional care. We only included studies if full text publications were available.

### Exclusion criteria

Publications were excluded if it concerned qualitative research, reviews, guidelines, case studies, editorials and abstracts.

## Study selection

Two reviewers independently screened all titles and abstracts to identify potentially relevant studies based on the inclusion criteria. If they could not assess a publication for relevance based on title and abstract, full text was obtained. Subsequently, the two reviewers independently studied the full texts for final inclusion. We used snowball sampling by hand-searched the reference lists of all included articles and relevant (systematic) reviews to identify additional publications. After each step, the reviewers discussed their findings to reach consensus. When disagreement needed to be solved, a third reviewer was consulted.

## Data extraction

Data were extracted by one reviewer and verified by a second reviewer. A self-designed extraction form was used. This form was pilot tested by two reviewers by comparing the data extraction results of the first 10 included studies. The following data were systematically collected: first author, publication year, country or origin, design of the study, setting, sample size, medical complexity of the child, age of the child, hospital-to-home intervention studied, outcomes, outcome measures, sources of the outcomes, and the time-frame of the outcome measures. Any outcomes that were described in the studies were included, and no distinction was made between primary and secondary outcomes.

## Analysis

A narrative synthesis was undertaken to summarize the outcomes. We anticipated that outcomes would vary in terminology and in measurement tools used. We merged the outcomes with similar definitions and/or concepts. Therefore, two researchers independently reviewed the list of outcomes as reported in the studies to identify similarities. Their findings were discussed in consensus meetings with the multidisciplinary hospital-to-home research group that included pediatricians, a pediatric intensivist, a pediatric rehabilitation specialist, (pediatric) nurses, and a clinical epidemiologist. Two researchers checked the merged outcomes by re-reading the publications.

After comparing several frameworks to classify the outcomes, we decided to use the taxonomy of Dodd et al. [23]. Based on this taxonomy, we categorized the outcomes into the following domains: (a) mortality and survival, (b) physical health, (c) life impact, (d) resource use, and (e) adverse events [23]. We created a domain (f) others to report on those outcomes that would not fit in well, but we considered important to include in this review. The domain mortality and survival includes all-cause mortality and cause-specific mortality. Physical health refers to measures of physiological function, signs and symptoms related to a body system, or general physiological outcomes, such as weight, fatigue and pain. Life impact refers to the impact of a disease or condition on functioning (e.g. social, emotional, and cognitive), quality of life, delivery of care (e.g. compliance), and personal circumstances (e.g. finances, work). Resource use includes outcomes related to healthcare utilization and costs. The domain adverse events includes any unintended consequence of the intervention. Two reviewers categorized the outcomes into one of the six domains. A third reviewer resolved uncertainties.

## Results

**Fig. 1:**
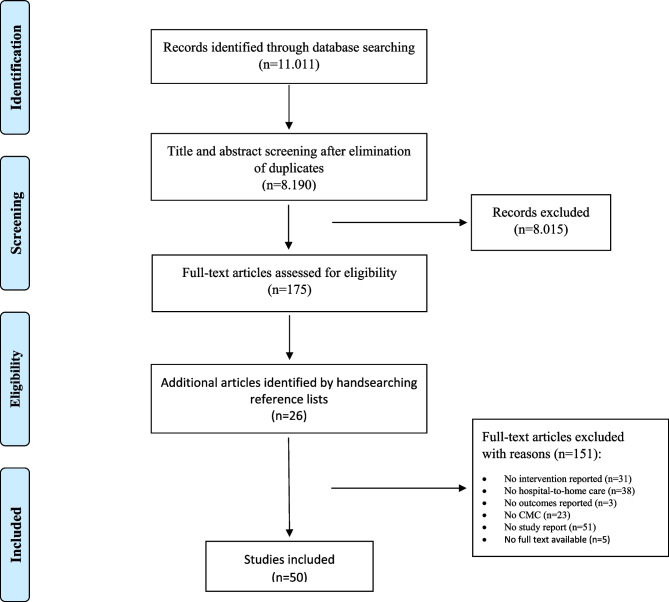
Study selection process

The database searches resulted in 11.011 records and after elimination of duplicates 8.190 records remained. A total of 8.015 papers were excluded based on title and abstract. After reading the remaining 175 publications full texts, 48 studies were deemed eligible according the pre-defined inclusion and exclusion criteria. Two additional publications were identified from hand searching the reference lists, resulting in a total of 50 studies included in this systematic review [24–73]. Figure 1 shows the selection process.

## Study characteristics

Of the 50 studies, 43 (86%) were performed in pediatric hospitals in the United States of America [24–28, 32–59, 61, 62, 64–70, 72], three studies in Canada (6%) [30, 31, 60], two in Italy (4%) [63, 73], one in Australia (2%) [29], and one in Turkey (2%) [71]. Fourty one studies (82%) were published between 2016 and 2021 [24–29, 32–38, 40, 42–47, 49–55, 59, 61–63, 65–70, 72, 73,57—]. In total, 29 studies (58%) characterized their study population as CMC, labeled with criteria, but no specific diagnosis [24, 25, 27–33, 35, 36, 38–42, 44, 46, 47, 49–52, 56, 59, 61, 63, 68, 73]. Other study populations were based on specific diagnoses: (preterm) neonates (six studies, 12%) [43, 48, 54, 66, 67, 70], neurological conditions or epilepsy (three studies, 6%) [34, 58, 60], and childhood cancer (one study, 2%) [71]. Some publications defined their population based on technology assistance: mechanical ventilation (two studies, 4%) [37, 53], tracheostomy (five studies, 10%) [45, 55, 57, 65, 72], or both (four studies, 8%) [26, 62, 64, 69].

The following designs were described in the publications: cohort studies (22 studies, 44%) [25, 27–29, 33, 35–37, 40, 41, 43, 53–56, 58, 59, 63, 66, 67, 70, 73], quality improvement projects with a pre-post design (13 studies, 26%) [24, 26, 38, 42, 45, 50, 51, 61, 62, 64, 65, 68, 72], randomized controlled trials (six studies, 12%) [32, 34, 46, 47, 49, 60], quasi-experimental studies (five studies, 10%) [39, 44, 48, 52, 71], mixed method studies (two studies, 4%) [30, 31], or other (two studies, 4%) [57, 69].

The hospital-to-home interventions studied were mainly multi-faceted including a wide variety of activities, e.g. care coordination, parental education programs, home visits, and telehealth applications. The study characteristics are summarized in Table 1.

**Table 1:** Characteristics of included studies

**Author (year)**	**Country**	**Study type**	**Setting**	**Sample size**	**Medical condition**	**Age of children**	**Intervention**	**Outcomes**
Antolick et al. (2020) [24]	USA	Quality improvement project with a pre-post design	Children's hospital	40	High risk children (criteria described)	Median 12 years	Post Hospitalization Action Grid (PHAG) and a standardized discharge handoff process.	- Provider and staff perceptions of integrated care - Provider and staff intentions to use the PHAG
Appachi et al. (2017) [25]	USA	Retrospective medical record review with a pre-post design	Tertiary Care Center for children with medical complexity	113	Children with complex medical needs, specifically with aerodigestive disorders (definition described)	Range 0 to 20 years	A multidisciplinary aerodigestive clinic providing a comprehensive and coordinated care program (inpatient and outpatient).	- Number of hospital admissions - Number of hospital days for all admissions per year - Number of admissions in the aerodigestive clinic - Costs
Baker et al. (2016) [26]	USA	Quality improvement project with a pre-post design	Tertiary care children's hospital	48	Children requiring chronic mechanical ventilation via tracheostomy	Neonates	An interdisciplinairy Ventilator Care Program including: education materials, a chronic ventilation road map and instructional videos for caregivers, assessment of discharge readiness, the involvement of an advanced practice nurse and rehabilitation respiratory therapist, and case management.	- Mortality - Number of unplanned hospital readmissions - Hospital length of stay - Number of Emergency Department visits - Costs
Barreda et al. (2021) [27]	USA	Propensity-matched retrospective cohort design	Children’s Hospital	566	Children with medical complexity (criteria described)	Median 5 years (IQR 4-6)	Pediatric Complex Care Program including: comprehensive care planning and care coordination, support by the complex care team for urgent visits, monthly phone calls.	- Number of hospitalisation per year, related to device complications - Number of Emergency Department visits per year, related to device complications
Braun et al. (2021) [28]	USA	Retrospective matched cohort design	Children’s Hospital	64	Children with medical complexity (criteria described)	Mean 5.6 years (SD 7.2) and 5.5 (SD 7.7)	Family Integrated Healthcare Transitions (FLIGHT) team providing care coordination and complex care by a predominantly telemedicine-mediated format.	- Number of hospital admissions per year - Hospital days per year and per admission - Number of subspeciality appointments per year - Missed appointments per year
Breen et al. (2018) [29]	Australia	Pre-post implementation cohort evaluation	Children’s Hospital Network (two tertiary pediatric hospitals)	534	Children with medical complexity (criteria described)	Median 5 years (range 7-19)	Care coordination service: access to a care coordinator, shared-care plan, linkage with local general practitioners, and acces to a 24-hours hotline.	- School absences child - Prevented family travel costs - Number of hospital encounters and admissions (overnight and day-only) - Number of Emergency Department visits - Costs
Cohen et al. (2010) [30]	Canada	Mixed method descriptive study with a pre-post design	Tertiary care hospital	28	Children with medical complexity (criteria described)	Median 1.5 years (range 30 days to 14 years)	Nurse practitioner/pediatrician-run complex care clinic in a tertiary care hospital.	- Quality of life of parents - Parental perceptions of care - Healthcare utilization (inpatient days, Emergency Department visits, visits to hospital and community based practitioners) - Healthcare providers perceptions of care
Cohen et al. (2012) [31]	Canada	Mixed method, intervention study with a pre-post design	A tertiary care children's hospital and two community hospitals	81	Children with medical complexity (criteria described)	Mean 5.8 years (SD 4.7)	Community-based complex care clinics integrated with a tertiary care center.	- Quality of life child - Quality of life of parents - Parental perception of family centered care - Health care utilization (inpatient days, Emergency Department visits, visits to hospital and community based practitioners) - Costs (healthcare system costs and parents reported out of pocket expenses)
Coller et al. (2018) [32]	USA	Randomized Controlled Trial	Tertiary medical center	147	Children with medical complexity	< 18 years	Plans for Action and Care Transition (PACT): written action plans and care transition coaching during the period of hospital discharge.	- Mortality - Number of readmissions - Number of hospitalizations - Costs
Donnelly et al. (2020) [33]	USA	Retrospective cohort study with a pre-post design	Tertiary children's hospital	31	Children with medical complexity (criteria described)	Mean 5.2 years (SD 3.5)	The Advanced Practice Nurse and Care Coordination Assistent model medical care coordination program.	- Quality of life child - Self-efficacy regarding managing child's healthcare needs - Quality of Life of parents - Satisfaction of parents with healthcare
Duffy and Vessey (2016) [34]	USA	Randomized Controlled Trial	Pediatric teaching hospital	46	Children with chronic neurological conditions	2 to 6 years	Creating Opportunities for Parent Empowerment (COPE) program: an intervention that teaches parents what behaviors they can expect in their child as normal response to illness, and how to help their child to cope with illness experiences.	- Number of behavior problems of the child - Parental belief in their caregiving skills - Anxiety of parents - Depression of parents
Gay et al. (2016) [35]	USA	Retrospective, matched cohort study	Home Health Care Nursing Care services across 19 states	10144	Children with medical complexity	0 to 18 years	Home Health service: intermittent skilled nursing visits or private duty nursing.	- Number of readmissions - Number of hospitalizations - Number of hospital days - Costs
Gillen and Morris (2019) [36]	USA	Cohort study with a pre-post design	Urban PICU	50	Children with medical complexity	< 18 years	Information and materials to improve the ability of caregivers to care for their children in case of a prolonged home power failure.	- Families' disaster preparedness
Graham et al. (2018) [37]	USA	Prospective cohort study	Tertiary care center	346	Children with chronic respiratory failure	Median 6 years (IQR 1 to 16)	Critical Care, Anesthesia, Perioperative Extension Program (CAPE): an individual tailored and comprehensive longitudinal service and home ventilator program.	- Number of hospitalizations - Number of Emergency Department visits - Costs
Hogan et al. (2022) [38]	USA	Pre-post design	Pediatric health system with inpatient and outpatient services	105	Children with medical complexity (criteria described)	90% of the children were < 10 years	The Compass Care Program, a consultative complex care program across inpatient and outpatient settings.	- Caregiver satisfaction (communication and access to care) - Number of hospital (re)admissions - Number of hospital readmissions within 7 days. - Number of hospital days - Length of stay per inpatient admission - Number of Emergency Department visits - Cost
Holland (2015) [39]	USA	Prospective quasi-experimental, non-equivalent comparison group design	Tertriary children's hospital	300	Pediatric patients hospitalized in the acute care setting	Mean 8.7 years (SD 5.9)	Early Screen for Discharge Planning Child Version (ESDP-C): a screening tool to identify children with medical complexity that would benefit from early engagement of a discharge planner.	- Time from admission to discharge planner involvement - Number of readmissions - Length of hospital stay
Howard et al. (2017) [40]	USA	Retrospective cohort study	A Pediatric Hospital and other urban hospitals	183	Children with medical complexity, including cerebral palsy	Mean 12.4 years (SD 5.8) and 6.7 (SD 4.4)	Care beyond The Bedside Model: increasing the preparedness and comfort level of children and their caregivers to manage ongoing health care needs independently.	- Number of readmissions - Number of inpatient days - Number of Emergency Department visits - Costs
Knight et al. (2013) [41]	USA	Observational survey-based study	Quarternary, academic pediatric hospital	117	High risk children (criteria described)	0 to 18 years	Cardiopulmonary resuscitation (CPR) discharge training.	- Parental knowledge of the core skills of CPR - Parents comfort levels in performing CPR - Frequency of video review and practising core skills after discharge by parents - Disemmination of the kit to other family and friends - Nurses' impression of the program and suggestions for improvement
Lerret et al. (2020) [42]	USA	Quality improvement project with a pre-post two group design	Academic medical center	395	Children with and without a chronic condition	2 to 21 years	The engaging Parents in Education for Discharge (ePed): a tool that explores parents experiences with discharge teaching and care coordination.	- Quality of discharge teaching - Quality of care coordination - Number of readmissions
Liu et al. (2018) [43]	USA	Cohort study with propensity score weighting	Tertiary Women and Infant hospital	686	High risk neonates (criteria are described)	0 to 1 year	Transition to Home Plus (THP) program: enhanced support services before and after discharge.	- Number of unplanned readmissions - Number of Emergency Department visits - Costs
Ming et al. (2022) [44]	USA	Non-randomized pilot study	Tertiary children’s hospital	48	Children with medical complexity (criteria described)	Mean 8.5 years (SD 5.6) and 10.1 years (SD 5.1)	Post-hospitalization telemedicine video visits during hospital-to-home transitions.	- Acceptability of the intervention (parents) - Saved travel costs - Self-efficacy - Number of hospital admissions - Length of hospital stay - Length of ICU stay - Number of Emergency Department visits
Moreno and Peck (2020) [45]	USA	Quality improvement project with a pre-post intervention design	Freestanding pediatric hospital	2	Children with a newly placed tracheostomy	Not reported	A hospital-based discharge protocol and telehealth follow-up care.	- Caregiver knowledge, competence, self-efficacy and satisfaction - Number of hospitalizations - Number of Emergency Room visits - Number of tracheostomy-associated complications
Mosquera et al. (2021a) [46]	USA	Randomized Quality Improvement Trial	High-Risk Children’s Clinic	422	Children with medical complexity (criteria described)	Mean 6.2 years (SD 5.4) and 5.7 years (SD 4.5)	Care coordination with telemedicine.	- Mortality - Serious illness - Hospital (re)admissions - Number of PICU admission - Number of hospital days - Hospital length of stay - PICU length of stay - Number of Emergency Department visits - Number of office visits - Well-child checks - Costs
Mosquera et al. (2021b) [47]	USA	Randomized Quality Improvement Trial	High-Risk Children’s Clinic	342	Children with medical complexity (criteria described)	Mean 5.8 years (SD 4.2) and 6.3 years (SD 4.7)	A hospital consultation service for CMC from their outpatient comprehensive care clinicians	- Mortality - Serious illness - Parental satisfaction healthcare - Hospital (re)admissions - Number of PICU admission - Number of hospital days - Hospital length of stay - PICU length of stay - Number of Emergency Department visits - Number of telephone conversations after discharge - Costs
Moyer et al. (2014) [48]	USA	Concurrent cohort study	Children's hospital	229	Preterm children (criteria described)	Preterm infants	Care Transitions Intervention: a care transition coach to assist families, and an enhanced personal health record to improve the quality of information available to parents and community professionals.	- Mortality - Number of sick visits - Number of unplanned readmissions - Number of Emergency Department visits - Caregiver perception of perceived quality of transitional care - Non-compliance with follow-up appointments
Nguyen et al. (2018) [50]	USA	Prospective cohort study	Freestanding pediatric hospital	311	Children with medical complexity (criteria described)	Range 0 to 17 years	Pharmacy discharge services additional to an existing nurse-led discharge service.	- Number and type of pharmacist interventions during discharge and telephone encounters - Costs
Nkoy et al. (2021) [49]	USA	Randomized controlled trial	Tertiary children’s hospital	50	Children with medical complexity (criteria described)	Mean 9.65 years (95% CI 7.40 to 11.91) and 7.29 years (95% CI 4.90 to 9.69)	A home monitoring application (mobile app) additional to the usual care coordination program.	- Health deteriorations - Quality of life child - Satisfaction with healthcare - Hospital admissions - Hospital days - Number of Emergency Department visits
Noritz et al. (2017) [51]	USA	Quality improvement project with a pre-post design	Freestanding quaternary children's hospital and the affiliated accountable care organizations	1070	Children with medical complexity (criteria described)	Range 1 to 18 years	Program with 3 interventions: standardizing percutaneous feeding tube management, improving family education, and care coordination.	- Number of children with a weight between 5th and 95th percentile on standard growth curve - Length of stay - Costs
Osorio et al. (2021) [52]	USA	Factorial design of a natural experiment	Four children’s hospitals	7725	Multiple or complex chronic conditions, including technology-supported patients	Median 1.9, 2.4, 0.7 and 3.6 years	Pediatric Care Transition (PACT) bundel: (a) a transition readiness checklist, (b) predischarge teach-back education, (c) written handoff to primary care professional, and (d) postdischarge phone call.	- Hospital readmission rate
Parker et al. (2020) [53]	USA	Cohort study with a comparison group	University-affiliated tertiary care children's hospital	126	Children with chronic respiratory insufficiency requiring technological support	Median 7 years (IQR 3 to 12)	The Center for Children with Complex and Chronic Conditions (C5) program: inpatient and outpatient care coordination.	- Number of readmissions - Number of Emergency Department visits - Costs
Patel et al. (2017) [54]	USA	Prospective observational cohort study	Regional NICU	241	High risk neonates (criteria described)	born < 32 weeks gestational age	BRIDGE: home visits by pediatric nurse practitioners.	- Number of adverse events: homecare and healthcare utilization errors, such as errors with medication, feeding and equiment use, - Non-compliance with follow-up appointments
Petitgout (2018) [55]	USA	Retrospective cohort study with a pre-post design	Tertiary children's hospital	158	Children with a tracheostomy	≤ 21 years	Hospital-based care coordination program: interdisciplinary family centered care, continuity of care, psychosocial support, assessment of recources and services, communication, avoidance of duplication of services and improving overall health of the child.	- Number of unplanned hospital readmissions - Length of stay - Costs
Postier et al. (2014) [56]	USA	Retrospective cohort study with a pre-post design	Pediatric hospital	425	Children with life-limiting and life-threatening illnesses	1 to 21 years	Pediatric Palliative Care program (PCC): a home-based pediatric hospice program.	- Number of hospital admissions - Length of stay - Costs
Prickett et al. (2019) [57]	USA	Evaluation study	Pediatric hospital	39	Children with a newly placed tracheostomy	Not reported	Tracheostomy simulation-based education program for caregivers: classroom learning, one-on-one teaching, bedside teaching and caregivers skills demonstrations.	- Parental self confidence with tracheostomy emergency management - Utility of tracheostomy emergency management
Roundy et al. (2016) [58]	USA	Cohort study with historical controls	Tertiary children's hospital.	120	Children with epilepsy	0 to 18 years	Seizure Action Plan (SAP): information that might be forgotten and difficult to remember, and that would be helpful in a situation of breakthrough seizures or for determining timing of follow-up.	- Number of readmissions - Length of stay - Number of Emergency Department visits - Number of neurology follow-up clinic visits - Number of telephone calls to pediatric neurology offices
Sarik et al. (2018) [59]	USA	Retrospective review	Pediatric acute care setting in a Magnet designated hospital	398	Children with medical complexity (criteria described)	0 to 20 years	Patient navigation program: assessment of the readiness for discharge, planning of the sequential order of appointments, implementing stategies to help families succesfully care for their child.	- Number of readmissions - Rate of no-show at scheduled follow-up appointments
Sigalet et al. (2014) [60]	Canada	Randomized Controlled pilot Trial	Children's hospital	61	Children with acute seizure disorders	< 18 years	Simulation-based seizure management teachingprogram.	- Caregivers self-efficacy - Caregivers performance assessed by the trainer
Statile et al. (2016) [61]	USA	Quality improvement project	Freestanding pediatric hospital	227	Children with neurologic medical complexity (categories described)	Median 5.3 years (IQR 2.2 to 15.6)	Quality improvement interventions, such as defining medical discharge goals, admission order sets, care coordination rounds, needs assessment tool, and medication pathway.	- Number of children discharged within 2 hours of meeting medical discharge goals - Number of readmissions - Length of hospital stay
Thrasher et al (2018) [62]	USA	Quality improvement project	University-affiliated tertiary care children's hospital and regional referral centers	87	Children with a tracheostomy requiring long-term mechanical ventilation	Median 11.5 months (range 2 to 410)	Simulation training incorporated into a multimodal discharge preparedness training with instructional videos, printed handouts, scenario training, and video based debriefing.	- Number of readmissions
Tiozzo et al. (2022) [63]	Italy	Prospective cohort study	Pediatric hospital	310	Children with chronic diseases and complex therapeutic plans	0 to 18 years	A Medication Safety at Home cell-phone app.	- Number and features of out-of-hospital medication errors
Tofil (2013)	USA	Quality improvement project	Department of pediatric pulmonology	7	Children with a trachestomy and home ventilation	Range 15 months to 15 years	Home ventilator program with simulation training.	- Parents perceived preparedness and confidence to provide care to their child
Tolomeo et al (2017) [65]	USA	Quality improvement project	Pediatric Respiratory Care Unit	30	Children with a trachestomy	< 1 year	Standardizing the care and skills proficiency training for parents of infants with trachestomy tubes: welcome binder and educational material.	- Number of developmental interventions for infants with tracheostomy tubes - Length of stay
Vohr et al. (2017) [66]	USA	Prospective cohort study	NICU (level 3-4)	804	High risk / very low birth weight preterm infants	Born ≤ 37 weeks gestational age	Comprehensive transition home services: predischarged interventions (e.g. education, support to assess community resources) and postdischarge interventions (e.g. contact with primary care provider).	- Number of rehospitalization
Vohr et al. (2018) [67]	USA	Prospective cohort study	NICU (level 3-4)	804	High risk / very low birth weight preterm infants	Early, moderate and late preterm infants.	NICU transition support services: predischarged interventions (e.g. education, support to assess community resources) and postdischarge interventions (e.g. contact with primary care provider and visits to follow-up clinic).	- Number of Emergency Department visits
Wells et al. (2017) [68]	USA	Prospective cohort study	Children's hospital	38	Children with medical complexity (criteria described)	Median 6 years (IQR 2 to 18)	Postdischarge home visits.	- Unresolved health issues - Parental satisfaction with home visits - Postdischarge problems (number and type)
Whalen et al. (2020) [69]	USA	Descriptive quantitative design	Children's hospital	8	Childeren with a tracheostomy, some requiring mechanical ventilation	≤ 18 years	Parental Airway Assessment with Simulation program.	- Parent tracheostomy skills
Willard et al. (2018) [70]	USA	Prospective cohort study	NICU (quaternary level 4)	93	Children with medical surgical complexity	Infants	Postdischarge telemedicine visits, as additional to the existing discharge process (education and home care coordination).	- Caregivers knowledge and practice gaps uncovered - Caregiver perceived satisfaction
Yilmaz and Ozsoy (2010) [71]	Turkey	A quasi-experimental study	Pediatric oncology unit	49	Children with newly diagnosed cancer	Mean 8.7 years (SD 5.9) and 10.7 years (SD 4.2)	A discharge planning program, including discharge planning, discharge teaching, home visits and postdischarge telephone consultation.	- Physical care needs of the children (number and characteristics) - Number of readmissions Number of clinic visits
Yuen et al. (2021) [72]	USA	Pilot study with a pre-post design	Children’s hospital within a tertiary academic medical center	25	Children with tracheostomy	< 21 years	Simulation-based Discharge Program	- Caregivers’ comfort and confidence to perform care at home - Caregivers’ skills to provide care
Zanello et al. (2017) [73]	Italy	Prospective cohort study	Hospitals participating in the Special Needs Kids Research Project	61	Children with medical complexity (criteria described)	Mean 5.8 months (SD 11.8)	Family Pediatrician: care coordination by pediatricians in primary care.	- Patient's needs requiring care coordination - Activities by the Family Pediatricians (number and type) - Prevented healthcare utilization

*USA* United States of America, *IQR* Inter Quartile Range, *SD* Standard Deviation, *95% CI* 95% Confidence Interval

## Outcomes

We identified 172 outcomes among the 50 included studies. Our research group extensively reviewed and discussed the outcome list to identify those with similar definitions, wording or meaning. Finally, consensus was reached on a list of 25 unique outcomes that were assigned to the six outcome domains. We present the outcomes per study (Table 1) and per domain (Table 2).

**Table 2:** Outcomes per domain

**Outcome**	**Data sources**	**References**
**Domain 1: mortality and survival (1 outcome)**		
Mortality (five studies, 10%)	Data from institutional databases or electronic medical records	Baker et al. [26], Coller et al. [32], Mosquera et al. [46, 47], Moyer et al. [48]
**Domain 2: physical health (1 outcome)**		
Disease management, in terms of e.g. serious illness, health deteriorations, or physical care needs (seven studies, 14%)	Electronic medical records, e.g. growth curve, development assessments, unresolved health issues Mobile app for monitoring child’s vital signs and symptom Children's Physical Care Needs (CPCN) measurement tool	Mosquera [46, 47], Nkoy et al [49], Noritz et al. [51], Tolomeo et al. [65], Wells et al. [68], Yilmaz and Ozsoy [71]
**Domain 3: life impact (13 outcomes)**		
***Outcomes reflecting the impact on the life of the child***		
Quality of Life (three studies, 6%)	Measurement tools: Child Health-Related Quality of Life (PedsQL) Caregiver Priorities and Child Health Index of Life with Disabilities (CPCHILD) A self-structured questionnaire based on existing validated surveys: Medical Home Family Index and the Client Perception of Coordination Questionnaire (CPCQ) Adapted survey based on Parent Perceptions of quality of life and healthcare satisfaction for CMC	Cohen et al. [31], Donnelly et al. [33], Nkoy et al. [49]
Behavioral problems (one study, 2%)	Measurement tool: Behavior Assessment System for Children - Parent Report Scale (BASC2-PRS)	Duffy and Vessey [34]
School absences among children agedover 5 years (one study, 2%)	Data collected by care coordinators on prevented hospital visits	Breen et al. [29]
***Outcomes reflecting the impact on the life of the parents***		
Self-efficacy (nine studies, 18%)	Measurement tools: Parental Beliefs Scale A self-structured questionnaire based on existing validated surveys: Medical Home Family Index and the Client Perception of Coordination Questionnaire (CPCQ) KidSIM-ASPIRE Parent Seizure Self-efficacy Questionnaire Care Transitions Measure Survey (CTMS) CPR Skill Competence Checklist 10-point rating scale Caregiving self-efficacy (CSE) Family self-management (FSM) self-constructed survey/assessment tool	Donnelly et al. [33], Duffy and Vessey [34], Knight et al. [41], Ming et al. [44], Moreno and Peck [45], Prickett et al. [57], Sigalet et al. [60], Tofil et al. [64], Yuen et al. [72]
The competency of parents to provide care for their child (eight studies, 16%)	Measurement tools: Parents Airway Assessment with Simulation skill checklists Caregiver Knowledge Checklist (CKC) the KidSIM-ASPIRE Emergent Seizure Management Checklist CPR Skill Competence Checklist self-constructed survey / assessment tool	Gillen and Morris [36], Knight et al. [41], Moreno and Peck [45], Prickett et al. [57], Sigalet et al. [60], Whalen et al. [69], Willard et al. [70], Yuen et al. [72]
Satisfaction with hospital-to-home transitional care (eight studies, 16%)	Measurement tools: Self-structured questionnaire based on existing validated surveys: Medical Home Family Index and the Client Perception of Coordination Questionnaire (CPCQ) Telehealth Satisfaction Survey (TSS) Care Transition Measure (CTM) Care Transition Measure neo (CTM Neo) delivery subscale of the Quality of Discharge Teaching Scale (QDTS-D) Self-constructed survey / assessment tool Telehealth Usability Questionnaire Survey adapted from: (a) the Consumer Assessment of Healthcare Providers and Systems Patient-Centered Medical Home survey and b) Medicare Coordinated Care Demonstration survey.	Donnelly et al. [33], Hogan et al. [38], Lerret et al. [42], Ming et al. [44], Moreno and Peck [45], Moyer et al. [48], Wells et al. [68], Willard et al. [70]
Compliance, in term of missed appointments to an outpatient department/clinic/subspecialist (four studies, 8%)	Data from institutional databases or electronic medical records	Braun et al. [28], Moyer et al. [48], Patel et al. [54], Sarik et al. [59]
Quality of Life (three studies, 6%)	Measurement tools: Parental Health-Related Quality of Life (SF-36) A self-structured questionnaire based on existing validated surveys: Medical Home Family Index and the Client Perception of Coordination Questionnaire (CPCQ)	Cohen et al. [30], Cohen et al. [31], Donnelly et al. [33]
Satisfaction with healthcare in general (three studies, 6%)	Measurement tool: Larsen's Client Satisfaction Questionnaire (LCSQ) Consumer Assessment of Healthcare Providers and Systems (CAHPS) Adapted survey based on the Client Satisfaction Questionnaire	Cohen et al. [30], Mosquera et al. [47], Nkoy et al. [49]
Out-of-pocket expenses (three study, 6%)	Measurement tool: Health and Social Service Utilization Questionnaire for expenditures: parents reported out of pocket expenses Travel costs savings estimated from prevented hospital visits	Breen et al. [29], Cohen et al. [31], Ming et al. [44]
Satisfaction with family centered care (two studies, 4%)	Measurement tool: Measures of Processes of Care (MPOC)	Cohen et al. [30], Cohen et al. [31]
Anxiety (one study, 2%)	Measurement tool: State-Trait Anxiety Inventory (STAI-Y)	Duffy and Vessey [34]
Depression (one study, 2%)	Measurement tool: Beck Depression Inventory II (BDI-11)	Duffy and Vessey [34]
**Domain 4: Resource use (8 outcomes)**		
Hospital (re)admissions (30 studies, 60%)	Measurement tool: Adapted Care Coordination Measurement Tool (CCMT) Data from institutional databases or electronic medical records	Appachi et al. [25], Baker et al. [26], Barreda et al. [27], Braun et al. [28], Breen et al. [29], Coller et al. [32], Gay et al. [35], Graham et al. [37], Hogan et al. [38], Holland et al. [39], Howard et al. [40], Lerret et al. [42], Liu et al. [43], Ming et al. [44], Moreno and Peck [45], Mosquera et al. [46, 47], Moyer et al. [48], Nkoy et al. [49], Osorio et al. [52], Parker et al. [53], Petitgout [55], Postier et al. [56], Roundy et al. [58], Sarik et al. [59], Statile et al. [61], Thrasher et al. [62], Vohr et al. [70],Yilmaz et al. [71], Zanello et al. [73]
Length of hospital stay (19 studies, 38%)	Data from institutional databases or electronic medical records	Appachi et al. [25], Baker et al. [26], Braun et al. [28], Cohen et al. [30], Cohen et al. [31], Gay et al. [35], Hogan et al. [38], Holland et al. [39], Howard et al. [40], Ming et al. [44], Mosquera et al. [46, 47], Nkoy et al. [49], Noritz et al. [51], Petitgout [55], Postier et al. [56], Roundy et al. [58], Statile et al. [61], Tolomeo et al. [65]
Number of visits Emergency Department (19 studies, 38%)	Data from institutional databases or electronic medical records	Baker et al. [26], Barreda et al. [27], Breen et al. [29], Cohen et al. [30], Cohen et al. [31], Graham et al. [37], Hogan et al. [38], Howard et al. [40], Liu et al. [43], Ming et al. [44], Moreno and Peck [45], Mosquera et al. [46, 47], Moyer et al. [48], Nkoy et al. [49], Parker et al. [53], Roundy et al. [58], Vohr et al. [66], Zanello et al. [73]
Costs (17 studies, 34%)	Data from institutional databases or insurance databases	Appachi et al. [25], Baker et al. [26], Breen et al. [29], Cohen et al. [31], Coller et al. [32], Gay et al. [35], Graham et al. [37], Hogan et al. [38], Howard [40], Liu et al. [43], Mosquera et al. [46, 50], Noritz et al. [51], Parker et al. [53], Petitgout [55], Postier et al. [56]
Number of contacts to an outpatient department/clinic/ subspecialist (nine studies, 18%)	Data from institutional databases or electronic medical records	Braun et al. [28], Breen et al. [29], Cohen et al. [31], Mosquera et al. [46, 47], Roundy et al. [58], Yilmaz and Ozsoy [71], Zanello et al. [73]
Number of primary care consultations or visits to a community based clinic (two studies, 4%)	Data from institutional databases or electronic medical records	Cohen et al. [30], Cohen et al. [31]
Services carried out by a pharmacist (one study, 2%)	Electronic medical records and a self-constructed tool	Nguyen et al. [50]
Number of activities performed by primary care professionals, e.g. laboratory tests, examinations, coordination services (one study, 2%)	Measurement tool: Special Needs Kids-Family Pediatrician (SpeNK-FP)	Zanello et al. [73]
**Domain 5: adverse events (1 outcome)**		
Identification, number and features of out-of-hospital medication and equipment errors (four studies, 8%)	Data collected by care coordinators on adverse events at home during home visits. Home cell-phone app	Moreno and Peck [45], Patel et al. [54], Tiozzo et al. [63], Wells et al. [68]
**Other: Staff perceptions (1 outcome)**		
Staff perception about the transitional care, in term of feasibility, usability and satisfaction (three studies, 6%)	Provider and Staff Perceptions of Integrated Care Survey Self-constructed survey	Antolick et al. [24], Cohen et al. [31], Knight et al. [41]

### Mortality and survival

Mortality was considered as an outcome in five studies (10%) [26, 32, 46–48]. Mortality was reported differently: 1-year mortality [26], number of children deceased during the study period [32, 46, 47], and 30-days mortality [48].

### Physical Health

In seven studies (14%) the outcomes referred to disease management [46, 47, 49, 51, 65, 68, 71]. Studies reported on different outcomes: serious illness [46, 47], health deterioration [49], weight on standard growth curve [51], physical development assessments [65], unresolved health issues [68], and physiological care needs, e.g. bowel control and pain [71].

### Life impact

This domain was evaluated in 24 studies (48%) [28–31, 33, 34, 36, 38, 41, 42, 44, 45, 47–49, 54, 57, 59, 60, 64, 47–70, 72] and we differentiated 13 outcomes. Five studies reported on outcomes reflecting the impact on the life of the child: quality of life (three studies, 6%) [31, 33, 49], behavioral problems (one study, 2%) [34], and school absences (one study, 2%) [29]. Ten outcomes concerned the impact on the lives of the parents: self-efficacy (nine studies, 18%) [33, 34, 41, 44, 45, 57, 60, 64, 72], the competency of parents to provide care for their child (eight studies, 16%) [36, 41, 45, 57, 60, 69, 70, 72], satisfaction with hospital-to-home transitional care (eight studies, 16%) [33, 38, 42, 44, 45, 48, 68, 70], compliance in terms of missed appointments were explored in four studies (8%) [28, 48, 54, 59], quality of life (three studies, 6%) [30, 31, 33], satisfaction with healthcare in general (three studies, 6%) [30, 47, 49], out-of-pocket expenses (three studies, 6%) [29, 30, 44], satisfaction with family centered care (two studies, 4%) [30, 31], anxiety (one study, 2%) [34], and depression (one study, 2%) [34].

### Resource use

The majority of the studies (36 studies, 72%) had chosen outcomes in the domain resource use [25–32, 35, 37–40, 42–53, 55, 56, 58, 59, 61, 62, 65–67, 71, 73]. Hospital (re)admission was the most frequently reported outcome (30 studies, 60%) [25–29, 32, 35, 37–40, 42–49, 52, 53, 55, 56, 58, 59, 61, 62, 66, 71, 73], followed by length of stay in the hospital (19 studies, 38%) [25, 26, 28, 30, 31, 35, 38–40, 44, 46, 47, 49, 51, 55, 56, 58, 61, 65], the number of visits to an Emergency Department (19 studies, 38%) [26, 27, 29–31, 37, 38, 40, 43–49, 53, 58, 67, 73], and costs (17 studies, 34%) [25, 26, 29, 31, 32, 35, 37, 38, 40, 43, 46, 47, 50, 51, 53, 55, 56]. Other reported outcomes in this domain were: the number of contacts to an outpatient department/clinic/subspecialist (nine studies, 18%) [28, 31, 46, 47, 58, 71, 73], the number of primary care consultations or visits to a community based clinic (two studies, 4%) [30, 31], services carried out by a pharmacist (one study, 2%) [50], and the number of activities performed by primary care professionals (one study, 2%) [73].

### Adverse events

Adverse events in terms of numbers and features of medication and equipment errors at home were assessed in four studies (8%) [45, 54, 63, 68].

### Other

Staff perception about the transitional care, in term of feasibility, usability and satisfaction was evaluated in three studies (6%) [24, 30, 41].

## Sources of outcome data and outcome measurements

Different data sources and tools were used to evaluate the outcomes. All studies reporting on mortality and healthcare use collected their data from institutional databases, insurance databases, and electronic medical records. Physical health data came from electronic medical records, a telehealth application and a measurement tool. Outcomes on physical health, life impact, adverse events, and staff perception were measured by a big variety of questionnaires or assessment tools. For example, in the studies reporting on life impact, we found 35 different measurement tools, of which several were modified or self-structured. See Table 2.

## Period of outcome measurements

The total duration of the study periods varied from three months [24, 45] to 10 years or more [25, 35, 55, 56]. We found great variation in the frequencies and intervals of the outcome measurements. Some studies reported a single observation, while other studies collected outcomes biweekly, monthly, quarterly, six-monthly, or yearly. Some studies included the measurements over time in the analyses [30, 34, 36, 43, 51, 59, 67, 71] and/or gave visual insight in the trends, e.g. with run charts [26, 29, 31, 49, 50, 54, 59, 61]. In general, studies were unclear in reporting their timelines.

## Discussion

In this systematic review, we identified outcomes currently reported in publications evaluating the effectiveness of hospital-to-home transitional care interventions for CMC. Despite a substantial degree of heterogeneity in the definitions and descriptions of the outcomes, we agreed on 25 unique outcomes. These outcomes were assigned to six main outcome domains: mortality and survival, physical health, life impact, resource use, adverse events, and others. This overview of outcomes shows the outcomes researchers have prioritized to evaluate hospital-to-home interventions.

We are aware of important previous work by Looman et al. [17] and Barnert et al. [74] that also reports on outcome measures in publications concerning CMC and their families. Looman et al. aim to identify patterns and gaps in classification systems, data, and outcomes in studies of CMC [17]. Barnert et al. aim to contribute to the development of summary measures to describe the health of CMC [74]. Our systematic review is of additional value due to its focus on outcomes used in evaluations of hospital-to-home interventions. In addition, we specifically aim to use this systematic review in the development of a COS in order to standardize and prioritize meaningful outcomes in studies that aim to improve hospital-to-home transitional care interventions for CMC and their families.

Most studies in our review had chosen resource use outcomes, such as visits to an ED, number of hospital admissions, length of hospital stay, and costs, which is congruent with the two aforementioned reviews [17, 74]. The focus on resource use outcomes might reflect the perceived importance by different stakeholders, e.g. policy makers, insurance companies, and healthcare professionals. The importance of resource use outcomes is obvious, as CMC account substantially in healthcare resource use, such as 20% of ED visits at children’s hospitals, and up to 33% of all children’s healthcare costs [4, 75, 76]. Furthermore, resource use outcomes might also be chosen as indicators of physical health, and the extent to which the medical complexity impacts on the child, parents and families.

Many studies in our systematic review had chosen outcomes reflecting the life impact. Within the domain life impact a variety of different outcomes were collected, of which most reflected the impact on the life of parents. The impact on the life of the children was less represented with six studies focusing on the child. We did not find studies reporting on the specific impact on siblings, other members of the family (e.g. grandparents), or family’s interactions. An explanation could be that those themes are more often explored in qualitative studies. To create a comprehensive view of hospital-to-home transitional care another review of qualitative studies should be of additional value. Thomson et al. pointed out that having a CMC may have a major negative impact on the financial situation of a family [8]. This is supported by Barnert et al. who underlined that a comprehensive and continuous health insurance is considered an important contribution to the health of CMC [77]. Apart from three study that reported out-of-pockets expenses [29, 31, 44], no other study in our review reported the financial impact as an outcome. This is in line with a recent review showing that out of 27 studies only three reported on costs of CMC from the family perspective [78].

Outcomes in the domains mortality and survival, and physical health were less represented in our results. It can be reasoned that the outcomes on physical health are associated with resource use, and therefore less chosen. For example, it is likely that disease exacerbations (physical health) result in more ED visits and hospitalizations (resource use outcomes), making resource use outcomes surrogate outcomes for physical health. We identified two studies that explicitly described the seriousness of the disease in terms of death and resource use [46, 47].

Adverse events outcomes were reported in only a few studies. This might be explained by the challenges in identification and reporting. The identification of errors at home depends mainly on self-reporting by parents. Lack of awareness, parents’ perceived value and decision-making of reporting, and non-transparent reporting processes might hamper data in this outcome domain [79]. However, minimizing medical errors was defined as an important outcome for a healthy life for CMC [77].

### Population

Although abundant literature exists on CMC, a uniform definition of CMC is lacking [80], and studies might not have been indexed clearly in the literature databases. Therefore, we also included studies that described the participants as children with complex chronic conditions. As a result, the studies included in this review represent a great variety of medical conditions and diseases, and over-inclusion in our study cannot be ruled out. As the aim of this SR was to identify all outcomes relevant for the hospital-to-home transition, we consider the broad inclusion as a strength of this study. On the other hand, also under-inclusion might have occurred as we excluded studies that did not provide a definition or clear description of the medical complexity of the participants. It is remarkable that most included studies are conducted in the USA. As outcome priorities may be influenced by national policy and the organization of care, this may have resulted in missing outcomes considered relevant in other countries.

### Period of outcome measurements

Obviously, the timing of outcome measurement in the course of disease is crucial and dependent on the aim of a study. Publication guidelines stress out to publish the rationale for the frequency, time between measurements and duration of the follow-up in studies. Especially in complex care, outcomes should not be considered final endpoints, but rather ongoing indicators of the well-being of patients and their families, and healthcare needs [81, 82]. It can be expected that the outcomes change over time influenced by the dynamic and unpredictable course of the condition of CMC, and the context. Longitudinal measurement should be encouraged, as it provides the opportunity to use results in feedback loops evaluating the impact and connecting the results with treatment, care, and support activities [82, 83].

### Limitations

We acknowledge some limitations of this systematic review. Firstly, we did not execute a quality assessment of the studies included in this systematic review. Because the aim of this systematic review was to summarize and categorize the reported outcomes regardless the quality of the study, we believe a quality assessment was not appropriate to address the research question of interest. However, future review of the existing literature is needed to quantify the effects of the interventions, including an assessment of the quality of the evidence, as this will inform the trustworthiness of the reported effectivity of the hospital-to-home interventions. Secondly, the final set of unique outcomes was established by consensus among the researchers. Despite the expertise of the researchers and the conscientious and careful process, subjective interpretations might have been of influence. In future research, validation by an independent group of experts might increase the trustworthiness of the results.

## Conclusion

This systematic review found a big variety of outcomes used in studies evaluating interventions to improve hospital-to-home transition for CMC. However, it was possible to summarize them in a short-list with 25 unique outcomes that reflect mortality, physical health, impact on the life of the child and the parents, resource use, adverse events and staff perceptions. This short-list may support researchers and program evaluators in outcome selection, and can be used in the development of a core outcome set transitional care for CMC in future.

### Supplementary Information

Below is the link to the electronic supplementary material.

Supplementary file1 (DOCX 37 KB)

## Data Availability

The datasets generated during and/or analyzed during the current study are available from the corresponding author upon reasonable request.

## Abbreviations

CMC: Children with Medical Complexity
COS: Core Outcome Set
COMET: Core Outcome Measures in Effectiveness Trials
ED: Emergency Department
PICU: Pediatric Intensive Care Unit
PRISMA: Preferred Reporting Items for Systematic Reviews and Meta-Analyses
