# Supplementary material for: Hospital-to-home transitions for children with medical complexity: part 1, a systematic review of reported outcomes
Source: Eur J Pediatr. 2023 Jun 15;182(9):3805–31. doi: 10.1007/s00431-023-05050-9 (PMC10570194; doi:10.1007/s00431-023-05050-9)
Supplement: Supplementary file 1 — Supplementary file1 (DOCX 37 KB) [file 431_2023_5050_MOESM1_ESM.docx]

Appendix A: search strategies

| **MEDLINE** | |
| --- | --- |
| **#** | **Searches** |
| 1 | Transitional Care/ |
| 2 | (exp Intensive Care Units, Pediatric/ or Critical Illness/ or Hospitals, Pediatric/ or Child, Hospitalized/) and (Patient Discharge/ or "length of stay"/) |
| 3 | (co-ordinated service* or coordinated service* or chronic care model* or discharge medical complex*).ti,ab,kf. |
| 4 | ((transition* or discharg* or follow* or care coordination) adj6 (home* or hospital or complex care)).ti,ab,kf. |
| 5 | 1 or 2 or 3 or 4 |
| 6 | Disabled Children/ |
| 7 | Patient Discharge/ and Risk Assessment/ |
| 8 | (chronic* or comorbid* or co-morbid* or multiple morbidit* or special medical condition* or CSHCN or CSHCNs or CMC or yshcn* or CYSHCN or CYSHCNs or disabled child* or medical fragil*).ti,ab,kf. |
| 9 | ((medical* or need* or health or care or disease*) adj3 complex*).ti,ab,kf. |
| 10 | ((special or extensive or intensive or healthcare or health care) adj3 need*).ti,ab,kf. |
| 11 | (technol* adj3 depend*).ti,ab,kf. |
| 12 | ((moderate or severe) adj3 disabilit*).ti,ab,kf. |
| 13 | 6 or 7 or 8 or 9 or 10 or 11 or 12 |
| 14 | exp Pediatrics/ or exp Infant/ or exp Child/ or Adolescent/ |
| 15 | (child* or infan* or neonat* or newborn* or premature or baby or babies or preschool* or pre-school* or preteen* or schoolchild* or pediatric* or paediatric* or juvenile or toddler* or kids or girl* or boy* or minors).ti,ab,kf. |
| 16 | 14 or 15 |
| 17 | 5 and 13 and 16 |
| 18 | comment/ or editorial/ or letter/ or news/ or (letter or comment* or editorial).ti. |
| 19 | (Adult/ or adult.ti. or ageing.ti,ab,kw.) not Child/ |
| 20 | ((pediatric or paediatric or transition* or transfer*) adj3 (to adult care or to adult service* or to adulthood)).ti,ab,kw. |
| 21 | 19 or 20 |
| 22 | 17 not 18 not 21 |
| 23 | limit 22 to yr="2010 -Current" |
| 24 | ((exp Animals/ or exp Animal Experimentation/ or exp models, animal/ or (animal* or rat or rats or mice or mouse or dog or dogs or pig or pigs or swine or swines or cow or cows or monkey or monkeys or goat or goats or horse or horses).ti,ab,kw.) not (Humans/ or human*.ti,ab,kw.)) or animal*.ti. |
| 25 | 23 not 24 |

| **EMBASE** | |
| --- | --- |
| **#** | **Searches** |
| 1 | transitional care/ |
| 2 | (pediatric intensive care unit/ or neonatal intensive care unit/ or critical illness/ or hospitalized child/ or child hospitalization/ or childhood disease/ or pediatric intensive care nursing/) and "length of stay"/ |
| 3 | (co-ordinated service* or coordinated service* or chronic care model* or discharge medical complex*).ti,ab,kw. |
| 4 | ((transition* or discharg* or follow* or care coordination) adj6 (home* or hospital or complex care)).ti,ab,kw. |
| 5 | 1 or 2 or 3 or 4 |
| 6 | handicapped child/ |
| 7 | hospital discharge/ and risk assessment/ |
| 8 | (chronic* or comorbid* or co-morbid* or multiple morbidit* or special medical condition* or CSHCN or CSHCNs or CMC or yshcn* or CYSHCN or CYSHCNs or disabled child* or medical fragil*).ti,ab,kw. |
| 9 | ((medical* or need* or health or health condition* or care or disease*) adj3 complex*).ti,ab,kw. |
| 10 | ((special or extensive or intensive or healthcare or health care) adj3 need*).ti,ab,kw. |
| 11 | (technol* adj3 depend*).ti,ab,kw. |
| 12 | ((moderate or severe) adj3 disabilit*).ti,ab,kw. |
| 13 | 6 or 7 or 8 or 9 or 10 or 11 or 12 |
| 14 | exp pediatrics/ or exp infant/ or exp child/ or adolescent/ or juvenile/ |
| 15 | (child* or infan* or neonat* or newborn* or premature or baby or babies or preschool* or pre-school* or preteen* or schoolchild* or pediatric* or paediatric* or juvenile or toddler* or kids or girl* or boy* or minors).ti,ab,kw. |
| 16 | 14 or 15 |
| 17 | 5 and 13 and 16 |
| 18 | letter/ or editorial/ or note/ or (letter or comment* or editorial).ti. |
| 19 | (adult/ or adult.ti. or ageing.ti,ab,kw.) not child/ |
| 20 | ((pediatric or paediatric or transition* or transfer*) adj3 (to adult care or to adult service* or to adulthood)).ti,ab,kw. |
| 21 | 19 or 20 |
| 22 | 17 not 18 not 21 |
| 23 | limit 22 to yr="2010 -Current" |
| 24 | limit 23 to conference abstract status |
| 25 | 23 not 24 |
| 26 | ((exp animal/ or exp animal experiment/ or exp animal model/ or (animal* or rat or rats or mice or mouse or dog or dogs or pig or pigs or swine or swines or cow or cows or monkey or monkeys or goat or goats or horse or horses).ti,ab,kw.) not (human/ or human*.ti,ab,kw.)) or animal*.ti. |
| 27 | 25 not 26 |

| **Cochrane Library** | |
| --- | --- |
| **#** | **Searches** |
| 1 | MeSH descriptor: [Transitional Care] explode all trees |
| 2 | (co-ordinated service* or coordinated service* or chronic care model* or discharge medical complex*):ti,ab,kw |
| 3 | ((transition* or discharg* or follow* or care coordination) near/3 (home* or hospital)):ti,ab,kw |
| 4 | ((pediatric intensive care unit* or critical illness or pediatric hospital or hospitalized child) and (patient discharge or length of stay)):ti,ab,kw |
| 5 | #1 or #2 or #3 or #4 |
| 6 | (chronic* or comorbid* or co-morbid* or multiple morbidit* or special medical condition* or CSHCN or CSHCNs or CMC or yshcn* or CYSHCN or CYSHCNs or disabled child* or medical fragil*):ti,ab,kw |
| 7 | MeSH descriptor: [Disabled Children] explode all trees |
| 8 | ((medical* or need* or health or health condition* or care or disease*) near/3 complex*):ti,ab,kw |
| 9 | ((special or extensive or intensive or healthcare or health care) near/3 need*):ti,ab,kw |
| 10 | (technol* near/3 depend*):ti,ab,kw |
| 11 | ((moderate or severe) near/3 disability):ti,ab,kw |
| 12 | #6 or #7 or #8 or #9 or #10 or #11 |
| 13 | (child* or infan* or neonat* or newborn* or premature or baby or babies or preschool* or pre-school* or preteen* or schoolchild* or pediatric* or paediatric* or juvenile or toddler* or kids or girl* or boy* or minors or adolescent*):ti,ab,kw |
| 14 | #5 and #12 and #13 with Cochrane Library publication date Between Jan 2010 and Jan 2020, in Cochrane Reviews, Trials |

| **PsycINFO** | |
| --- | --- |
| **#** | **Searches** |
| 1 | hospital discharge/ or discharge planning/ or health care services/ |
| 2 | exp Pediatrics/ and exp Intensive Care/ |
| 3 | (co-ordinated service* or coordinated service* or chronic care model* or discharge medical complex*).ti,ab,id. |
| 4 | ((transition* or discharg* or follow* or care coordination) adj6 (home* or hospital or complex care)).ti,ab,id. |
| 5 | 1 or 2 or 3 or 4 |
| 6 | (chronic* or comorbid* or co-morbid* or multiple morbidit* or special medical condition* or CSHCN or CSHCNs or CMC or yshcn* or CYSHCN or CYSHCNs or disabled child* or medical fragil*).ti,ab,id. |
| 7 | ((medical* or need* or health or care or disease*) adj3 complex*).ti,ab,id. |
| 8 | ((special or extensive or intensive or healthcare or health care) adj3 need*).ti,ab,id. |
| 9 | (technol* adj3 depend*).ti,ab,id. |
| 10 | ((moderate or severe) adj3 disabilit*).ti,ab,id. |
| 11 | exp multiple disabilities/ |
| 12 | 6 or 7 or 8 or 9 or 10 or 11 |
| 13 | pediatrics/ or chronically ill children/ |
| 14 | (child* or infan* or neonat* or newborn* or premature or baby or babies or preschool* or pre-school* or preteen* or schoolchild* or pediatric* or paediatric* or juvenile or toddler* or kids or girl* or boy* or minors).ti,ab,id. |
| 15 | 13 or 14 |
| 16 | 5 and 12 and 15 |
| 17 | exp aging/ or adult.ti. or ageing.ti,ab,id. |
| 18 | ((pediatric or paediatric or transition* or transfer*) adj3 (to adult care or to adult service* or to adulthood)).ti,ab,id. |
| 19 | 17 or 18 |
| 20 | 16 not 17 not 19 |
| 21 | limit 16 to yr="2010 -Current" |

| **CINAHL** | |
| --- | --- |
| **#** | **Searches** |
| 1 | MH "Transitional Care" OR ( ((MH "Intensive Care Units, Pediatric+") AND ( (MH "Length of Stay") OR (MH "Patient Discharge+") ) OR ( ( TI ( co-ordinated service* or coordinated service* or chronic care model* or discharge medical complex* ) OR AB ( co-ordinated service* or coordinated service* or chronic care model* or discharge medical complex* ) ) OR ( TI ( (transition* or discharg* or follow* or care coordination) N6 (home* or hospital) ) OR AB ( (transition* or discharg* or follow* or care coordination) N6 (home* or hospital) ) ) ) |
| 2 | MH "Child, Disabled" OR ( TI ( chronic* or comorbid* or co-morbid* or multiple morbidit* or special medical condition* or CSHCN or CSHCNs or CMC or yshcn* or CYSHCN or CYSHCNs or disabled child* or medical fragil* ) OR AB ( chronic* or comorbid* or co-morbid* or multiple morbidit* or special medical condition* or CSHCN or CSHCNs or CMC or yshcn* or CYSHCN or CYSHCNs or disabled child* or medical fragil* ) ) OR ( TI ( (special or extensive or intensive or healthcare or health care) N3 need* ) OR AB ( (special or extensive or intensive or healthcare or health care) N3 need* ) ) OR ( TI technol* N3 depend* OR AB technol* N3 depend* ) OR ( TI ( (moderate or severe) N1 disability ) OR AB ( (moderate or severe) N1 disability ) ) |
| 3 | (MH "Pediatrics+") OR (MH "Child+") OR (MH "Infant+") OR TI ( child* or infan* or neonat* or newborn* or premature or baby or babies or preschool* or pre-school* or preteen* or schoolchild* or pediatric* or paediatric* or juvenile or toddler* or kids or girl* or boy* or minors ) OR AB ( child* or infan* or neonat* or newborn* or premature or baby or babies or preschool* or pre-school* or preteen* or schoolchild* or pediatric* or paediatric* or juvenile or toddler* or kids or girl* or boy* or minors ) |
| 4 | 1 AND 2 AND 3 |
| 5 | ( (MH "Adult") OR TI adult ) NOT (MH "Child+") |
| 6 | TI ( (pediatric or paediatric or transition* or transfer*) N3 (to adult care or to adult service* or to adulthood) ) OR AB ( (pediatric or paediatric or transition* or transfer*) N3 (to adult care or to adult service* or to adulthood) ) |
| 7 | 4 NOT 5 NOT 6 |

| **WEB OF SCIENCE** | |
| --- | --- |
| **#** | **Searches** |
| 1 | **TOPIC:** ("complex chronic conditions" or "children with medical complexity" or technology dependen*) *AND* **TOPIC:** (child* or pediatric* or paediatric* or infant* or newborn* or baby or babies or youth) *NOT* **TITLE:** (adult) |
